# Supplementary material for: A New Pentafluorothio-Substituted Curcuminoid with Superior Antitumor Activity
Source: Biomolecules. 2021 Jun 25;11(7):947. doi: 10.3390/biom11070947 (PMC8301868; doi:10.3390/biom11070947)
Supplement: Supplementary file 1 [file biomolecules-11-00947-s001.zip › biomolecules-1253944-supplementary.pdf]

biomolecules

MDPI

Supporting Information

A New Pentafluorothio-Substituted Curcuminoid with Superior Antitumor Activity

Benedikt Linder [1,*,†], Leonhard H. F. Köhler [2,†], Lisa Reisbeck [1], Dominic Menger [1], Dharmalingam Subramaniam [3], Christel Herold-Mende [4], Shrikant Anant [3], Rainer Schobert [2], Bernhard Biersack [2,*] and Donat Kögel [1,5]

[1] Experimental Neurosurgery, Frankfurt University Hospital, Theodor-Stern-Kai 7, 60590 Frankfurt am Main, Germany; Lisa.Henkel@kgu.de (L.R.); dominic.menger@gmx.de (D.M.); koegel@em.uni-frankfurt.de (D.K.)
[2] Organic Chemistry Laboratory, University of Bayreuth, Universitätsstrasse 30, 95440 Bayreuth, Germany; Leonhard.Koehler@uni-bayreuth.de (L.H.F.K.); rainer.schobert@uni-bayreuth.de (R.S.)
[3] Cancer Biology Department, University of Kansas Medical Center, 3901 Rainbow Boulevard, Kansas City, MO 66160, USA; dsubramaniam@kumc.edu (D.S.); sanant@kumc.edu (S.A.)
[4] Department of Neurosurgery, Division of Experimental Neurosurgery, University Hospital Heidelberg, INF 400, 69120 Heidelberg, Germany; Christel.Herold-Mende@med.uni-heidelberg.de
[5] German Cancer Consortium (DKTK), Partner Site Frankfurt, 60590 Frankfurt am Main, Germany
* Correspondence: linder@med.uni-frankfurt.de (B.L.); bernhard.biersack@yahoo.com (B.B.)
† These authors have contributed to this work equally.

Citation: Linder, B.; Köhler, L.H.F.; Reisbeck, L.; Menger, D.; Subramaniam, D.; Herold-Mende, C.; Anant, S.; Schobert, R.; Biersack, B.; Kögel, D. A New Pentafluorothio-Substituted Curcuminoid with Superior Antitumor Activity. *Biomolecules* **2021**, *11*, 947. https://doi.org/10.3390/biom11070947

Academic Editor: Albino Martins

Received: 25 May 2021
Accepted: 22 June 2021
Published: 25 June 2021

Publisher's Note: MDPI stays neutral with regard to jurisdictional claims in published maps and institutional affiliations.

Copyright: © 2021 by the authors. Licensee MDPI, Basel, Switzerland. This article is an open access article distributed under the terms and conditions of the Creative Commons Attribution (CC BY) license (http://creativecommons.org/licenses/by/4.0/).

1. NMR-Stability Testing

The hydrolysis stability of substances **1a**, **1b** and **2** were monitored via ^{1}H NMR spectroscopy (500 MHz). The measurement was carried out in dimethylsulfoxide-d_6 and 5 vol-% D_2O for 0, 24, 48 and 72 h.

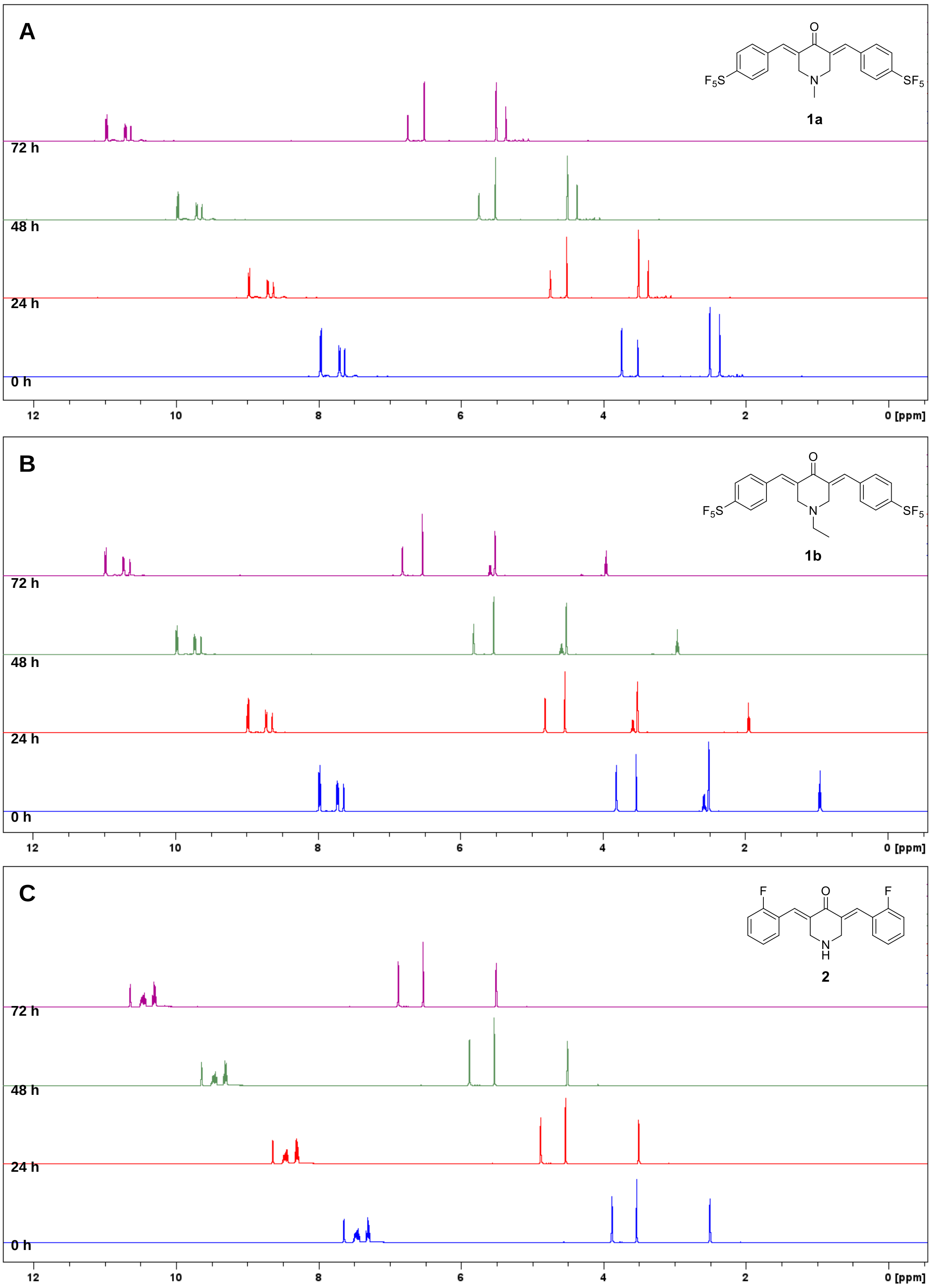

Figure S1. ^{1}H-NMR (500 MHz, dimethylsulfoxide-d_6, 5 vol-% water-d_2) spectra of **1a** (**A**), **1b** (**B**) and **2** (**C**), 0 h, 24 h (+2 ppm), 48 h (+4 ppm) and 72 h (+6 ppm) after preparing of stock solution.

2. Alteration in Cell-Cycle Progression of HT-29 Cells

The effect of compounds **1a**, **1b** and **2** (1 µM) on cell cycle distribution of HT-29 colon carcinoma cells were assessed via PI staining and subsequent flow cytometry (Figure S2).

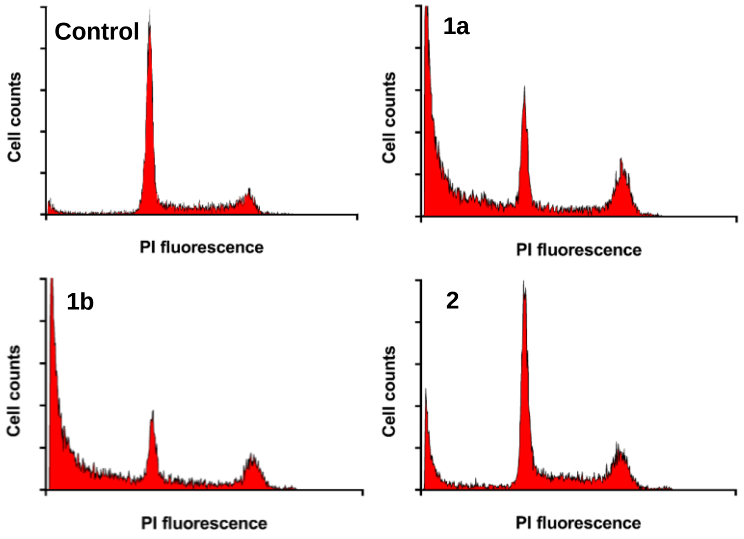

Figure S2. Representative cell cycle Histograms of HT-29 colon carcinoma cells treated with 1 µM of 1a, 1b and 2. Negative controls were treated with an equivalent amount of solvent (DMSO).

3. Activation of Executioner Caspases 3 and 7 in HT-29 Cells

As previous experiments showed that apoptosis induction might be one of the major effects of **1a**, **1b** and **2** we investigated the activity of effector caspases 3 and 7 through an Apo-One® Homogenous Caspase-3/7 Assay Kit (Fig. S3). The activity of caspases 3 and 7 was increased to 254 % after treatment with **1a**, 218 % for **1b** and 145 % for **2** after 6 h when compared with vehicle treated HT-29 cells. The positive control Staurosporine (STS) induced an increase in caspase activity of 442 % with a concentration of 2 µM.

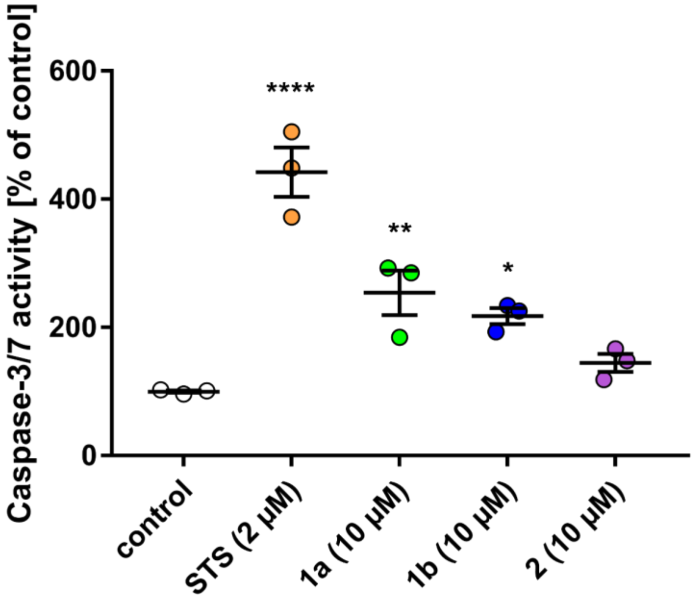

Figure S3. Measurement of caspase-3/7 activity via Apo-ONE® Homogenous Caspase-3/7 Assay Kit (Promega) after treatment of HT-29 colorectal adenocarcinoma cells with **1a**, **1b** and **2** (10 µm) for 6 h. 2 µM Staurosporine (STS) served as positive control, negative control was treated with an equivalent amount of solvent (DMSO). Cell vitality was reviewed via MTT assay and was offset against the values. The measurements were performed in triplicate and quoted as means ± SEM (n=3 per group). *: $p < 0.05$; **: $p < 0.01$; ***: $p < 0.001$; ****: $p<0.0001$, One-way ANOVA with Dunnett's multiple comparison test (GraphPad Prism 7).

4. Induction of Cell Death in GBM Cell Lines

The induction of early apoptosis and cell death by **1a** and **1b** in U251 and Mz54 GBM cells was evaluated by flow cytometry after staining with Annexin V-APC and PI. Representative dot plots of (Figure S4A) U251 and (Figure S4B) Mz54 GBM cells after treatment with solvent (DMSO), 0.3 µM **1a** and 0.5 µM **1b** is shown. Staurosporin (STS, 3µM) served as a positive control for potent cell death induction.

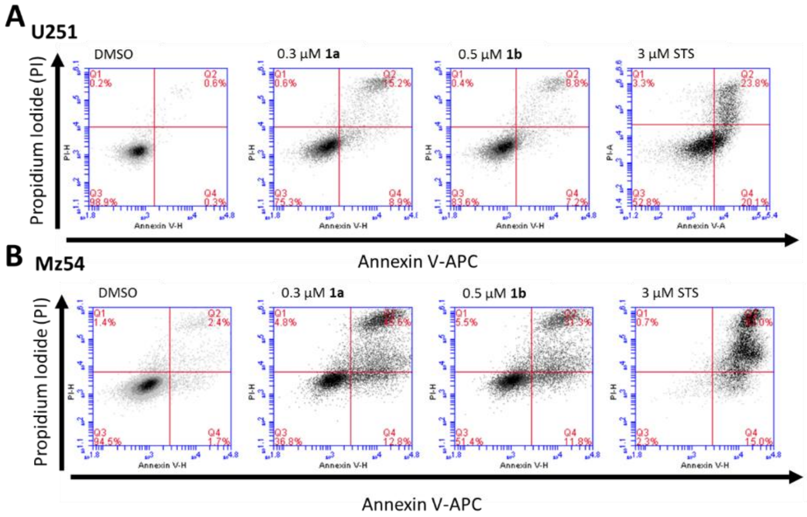

Figure S4. Concentration-dependent induction of cell death in conventional GBM cell lines U251-MG and Mz54 after treatment for 48h. FACS-based detection of Annexin V (An)- and Propidium Iodide (PI)-stained cells. (**A** and **B**) Example point-plots of (**A**) U251-MG (U251) and (**B**) Mz-54 (Mz54) after treatment (from left to right) solvent, DMSO, 0.3 µM **1a**, 0.5 µM **1b** or 3 µM Staurosporine (STS) as a positive control.
